# The Role of Thioredoxin Reductases in Brain Development

**DOI:** 10.1371/journal.pone.0001813

**Published:** 2008-03-19

**Authors:** Jonna Soerensen, Cemile Jakupoglu, Heike Beck, Heidi Förster, Jörg Schmidt, Wolfgang Schmahl, Ulrich Schweizer, Marcus Conrad, Markus Brielmeier

**Affiliations:** 1 Department of Comparative Medicine, Helmholtz Zentrum München German Research Center for Environmental Health, Neuherberg, Germany; 2 Institute of Clinical Molecular Biology and Tumour Genetics, Helmholtz Zentrum München German Research Center for Environmental Health, Munich, Germany; 3 AG Neurobiology of Selenium, Neuroscience Research Center and Institute for Experimental Endocrinology, Charité-Universitätsmedizin, Berlin, Germany; 4 Institute of Veterinary Pathology, Chair of General Pathology & Neuropathology, Ludwig-Maximilians University, Munich, Germany; 5 Institute of Physiology and Walter-Brendel Center, Ludwig-Maximilians University, Munich, Germany; National Institutes of Health/National Cancer Institute, United States of America

## Abstract

The thioredoxin-dependent system is an essential regulator of cellular redox balance. Since oxidative stress has been linked with neurodegenerative disease, we studied the roles of thioredoxin reductases in brain using mice with nervous system (NS)-specific deletion of cytosolic (*Txnrd1*) and mitochondrial (*Txnrd2*) thioredoxin reductase. While NS-specific *Txnrd2* null mice develop normally, mice lacking *Txnrd1* in the NS were significantly smaller and displayed ataxia and tremor. A striking patterned cerebellar hypoplasia was observed. Proliferation of the external granular layer (EGL) was strongly reduced and fissure formation and laminar organisation of the cerebellar cortex was impaired in the rostral portion of the cerebellum. Purkinje cells were ectopically located and their dendrites stunted. The Bergmann glial network was disorganized and showed a pronounced reduction in fiber strength. Cerebellar hypoplasia did not result from increased apoptosis, but from decreased proliferation of granule cell precursors within the EGL. Of note, neuron-specific inactivation of *Txnrd1* did not result in cerebellar hypoplasia, suggesting a vital role for *Txnrd1* in Bergmann glia or neuronal precursor cells.

## Introduction

The thioredoxin (Txn) dependent system is one of the key systems controlling cellular redox balance, and thus cell fate. Impaired cellular redox balance has been linked with age-related diseases, and neurodegenerative diseases, such as Alzheimer and Parkinson's disease [1]. Beyond its protective role, the Txn system is involved in various cellular processes, such as cell-cell communication, transcriptional regulation, cell signalling, and DNA synthesis [2].

The importance of the Txn system during embryo development and adult physiology has been corroborated by loss-of function approaches of individual members of this system in the mouse. Targeted inactivation of cytosolic thioredoxin (*Txn1*) was associated with severe defects of inner cell mass proliferation and embryonic death at the blastocysts stage [3]. Mice deficient for mitochondrial thioredoxin (*Txn2*) displayed marked apoptosis, anterior neural tube defects and embryonic lethality at E10.5 [4]. By contrast, overexpression of members of the thioredoxin family promoted neuronal survival after various stresses. For instance, *Txn1* overexpression in mice had a neuroprotective effect on neuronal cells when challenged with either focal brain ischemia [5] or with the excitotoxin kainate [6]. Systemic administration of recombinant human Txn1 decreased the extent of damage induced by focal brain ischemia [7].

Txn activities are governed by the selenoenzyme family of thioredoxin reductases [8]. There are three distinct genes in mammals encoding cytosolic thioredoxin reductase (*Txnrd1*)[9], mitochondrial thioredoxin reductase (*Txnrd2*)[10], and thioredoxin-glutaredoxin reductase (TGR or *Txnrd3*)[11]. While *Txnrd1* and *Txnrd2* are ubiquitously expressed in cells and tissues, expression of *Txrnd3* is mainly confined to testis. Thioredoxin reductases are flavoproteins, form homodimers in a head to tail alignment and possess two catalytically interacting reactive centres. The C-terminally located redox active site contains an essential selenocysteine residue; hence thioredoxin reductase activity relies on the availability of dietary selenium (Se). While some organs apparently exhibit a decreased demand for selenium, it is strongly retained in the brain during experimental selenium deficiency, arguing for essential roles exerted by one or several selenoproteins in this organ [12]. Accordingly, impaired expression of selenoproteins in transgenic mice caused developmental and degenerative damage in the brain [13]–[15].

Starting to dissect their contribution in embryonic development, adult physiology and pathophysiology, we created mice with conditional alleles of both *Txnrd1* and *Txnrd2* [16], [17]. Ubiquitous inactivation revealed that both genes are indispensable for embryogenesis albeit in different ways. *Txnrd1* knockouts exhibited remarkable developmental, growth and neural tube closure retardation and embryonic death between E8.5 and E10.5. *Txnrd2*-deficient embryos died shortly thereafter (E13.5) due to severe anemia and growth retardation, resulting from perturbed cardiac development and augmented apoptosis of hematopoietic cells.

Expression profiling of *Txnrd1* in embryos revealed highest expression levels in neuronal tissues such as the developing forebrain, rhombomeres and neural tube [17]. To ask whether thioredoxin reductases are essential in brain development and function, we inactivated both *Txnrd1* and *Txnrd2* individually in the nervous system (NS) by using the Nestin-Cre transgenic mouse [18], which expresses Cre in neuronal and glial precursor cells. NS-specific *Txnrd1* knockout mice were born at the expected frequencies, but displayed growth retardation and striking a movement disorder, suggestive of cerebellar dysfunction. By contrast, NS-specific ablation of *Txnrd2* or neuron-specific deletion of *Txnrd1* did not cause any obvious pathophysiological abnormalities.

## Results

### NS-specific *Txnrd1* knockout mice are smaller and display severe movement disorders

To decipher the individual contribution of cytosolic and mitochondrial thioredoxin reductase in brain development and maintenance, *Txnrd1* and *Txnrd2* expression in the central nervous system (CNS) was disrupted in a tissue-specific manner. To bypass embryonic lethality of *Txnrd1* and *Txnrd2* null embryos [16], [17], we used the *Nestin-Cre* (*NesCre*) transgenic mouse line, which targets precursor cells of the neuronal and glial lineage, thus achieving efficient gene deletion in both cell types [18], [19]. *Txnrd1*-NS null mice were born at the expected Mendelian ratio. Out of 197 offspring from *NesCre;Txnrd1^+/fl^* and *Txnrd1^fl/fl^* matings, 24% were *Txnrd1^+/fl^* , 24% were *Txnrd1^fl/fl^* , 22% were *NesCre;Txnrd1^+/fl^* and 28% were *NesCre;Txnrd1^fl/fl^*.(*Txnrd1*-NS null). Likewise, mice lacking *Txnrd2* specifically in the NS were born at the Mendelian ratio (data not shown).

**Figure 1:**
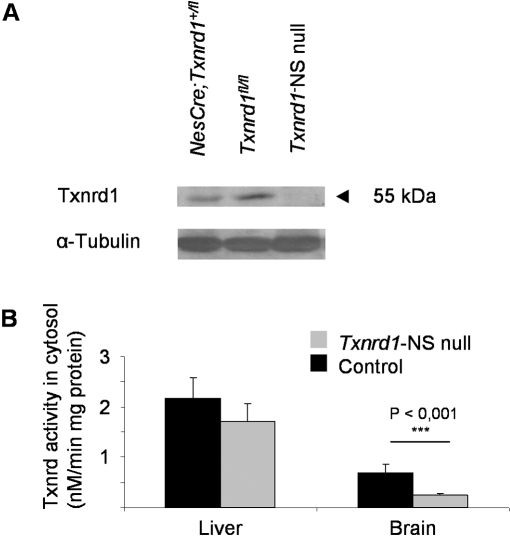
Txnrd1 expression is strongly reduced in *Txnrd1*-NS null mouse brain. (A) Western blot analysis of cytoplasmic brain protein extracts, revealing strongly reduced Txnrd1 protein levels in brain tissue of *Txnrd1*-NS null mice as compared to control mice. 30 µg protein were separated per lane. α-tubulin served as loading control. (B) Thioredoxin reductase activities were significantly reduced in the cytosol of *Txnrd1*-NS null brain but not of control brain nor of liver tissue regardless of genotype.

The efficiency of Cre-mediated gene deletion of *Txnrd1* and *Txnrd2* was monitored by immunoblotting, enzyme activity and qRT-PCR as shown in figure 1 and figure S1 A, respectively. While Txnrd1 protein levels in brain were only slightly reduced in *NesCre;Txnrd1^+/fl^* mice compared to *Txnrd1^fl/fl^* control mice, the 55-kDa immunoreactive band, representing Txnrd1 protein, was virtually absent in the brains of *Txnrd1*-NS null mice (Fig. 1 A). In accordance with the immunoblotting analysis, determination of cytosolic Txnrd activity in brain and liver tissue revealed significantly decreased cytosolic Txnrd activity in the brain of *Txnrd1*-NS null mice compared to control littermates (Fig. 1 B). qRT-PCR analysis of total RNA revealed very low levels of *Txnrd2* transcripts in brain of *Txnrd2*-NS null mice (Fig. S1 A).

**Figure 2:**
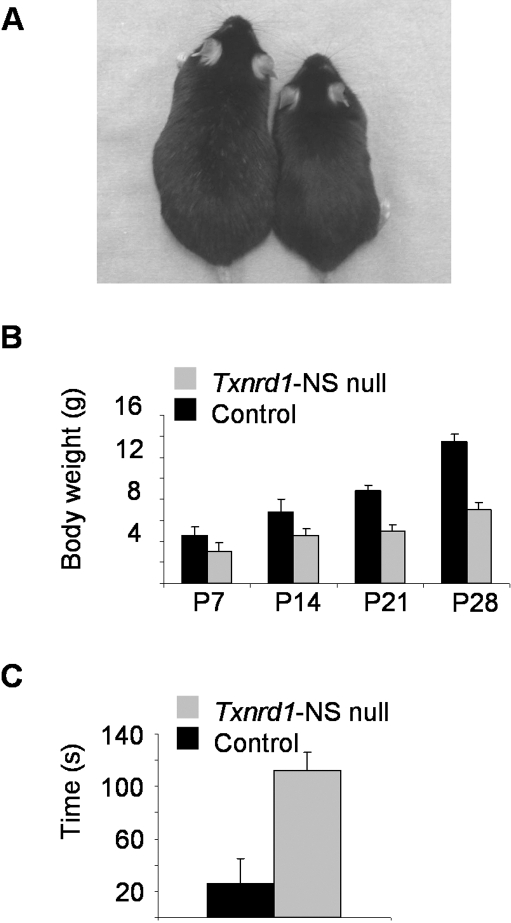
NS-specific *Txnrd1* knockout mice display restricted growth and striking movement disorders. (A) Shown are a *Txnrd1*-NS null mouse (right) and a control littermate (left) at the age of 1.5 years. (B) Determination of body weight at various time points revealed impaired growth of *Txnrd1*-NS null mice compared to control littermates. Values are expressed as mean±SD. (C) Motor coordination performance was tested using the pole test. Shown is the time required to climb down the vertical pole on 5 consecutive trials. If a mouse fell down, slipped or was unable to climb down, a default value of 120 s was taken into account. (P = postnatal day).

At birth, *Txnrd1*-NS null pups and *Txnrd2*-NS null pups were indistinguishable from their littermates, but within the first days of life, a deficiency in growth became apparent in *Txnrd1*-NS null mice, whereas *Txnrd2*-NS null mice developed normally and did not show any overt phenotype. NS-specific *Txnrd1* knockout mice slowly gained weight, reaching only about half the body mass compared to their littermates at the age of 4 weeks (Fig. 2 A and B). Already at P7, *Txnrd1*-NS null mice showed ataxic gait, impaired balance, and tremor. After weaning, NS-specific *Txnrd1* null mice were incapable of reaching food pellets in the wire bar of the cage. Only when supplied with food pellets on the bottom of the cage for the first couple of weeks, the knockout mice were able to grow to adulthood. Male and female knockout mice were fertile, and females could also foster their offspring normally (data not shown). In order to quantify the movement defect, *Txnrd1*-NS null and control mice were subjected to a modified pole test [20], an assay for coordinated movement (Fig. 2 C). While control animals were able to climb down the pole in approximately 30 seconds, *Txnrd1*-NS null mice mostly failed in doing so. 11 out of 14 tested mutants slid or dropped down.

### Cerebellar hypoplasia, abnormal foliation and perturbed lamination in the cerebellum of NS-specific *Txnrd1* knockout mice

**Figure 3:**
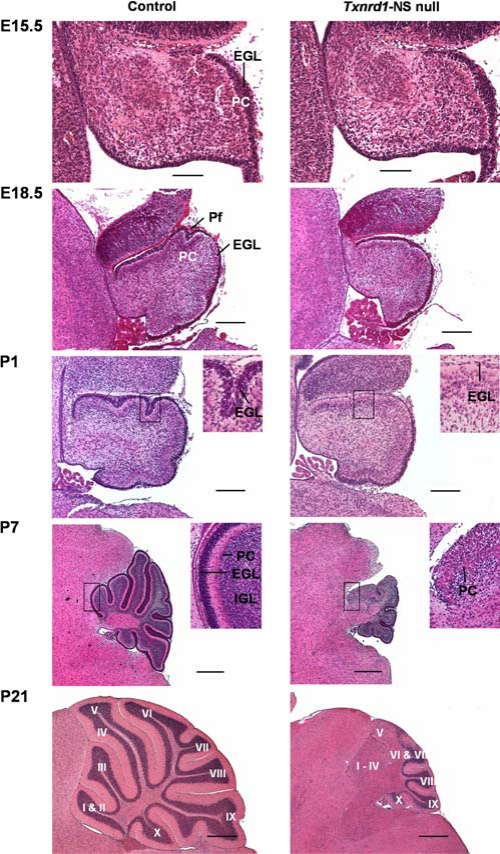
Striking cerebellar hypoplasia in *Txnrd1*-NS null mice. H&E staining of midline sagittal cerebellar sections from control (left column) and *Txnrd1*-NS null mice (right column) at embryonic stages E15.5, E18.5 and at postnatal days P1, P7 and P21 showed progressive differences in the foliation and formation of the molecular-, Purkinje cell- and granular layers during development of the cerebellum. In each photograph, the anterior part of the cerebellum is located to the left and the dorsal part to the top. At E15.5, no difference in cerebellar development is visible. At E18.5, the cerebellum is smaller and the formation of the primary fissure is slightly retarded in *Txnrd1* null brains. The external granular layer (EGL), which is the source of cells for the granular layer, is hypoplastic particularly in the anterior cerebellar area in *Txnrd1*-NS null mice at P1. Higher magnification demonstrated clear differences in the EGL thickness at P1 and P7 as well as the ectopic localisation of Purkinje cells and the absence of a well-organized, trilaminar-stuctured cerebellum. Already from E18.5 onwards, the *Txnrd1* null cerebella are smaller when compared to controls, and the laminar organisation of the mutant cerebellum becomes progressively more distorted in the anterior part. At P21, the posterior lobules X to VII of the knockout mice appear relatively well developed, whereas within lobules VI–V there is an abrupt transition from an apparently normally structured cortex to a disrupted cortex. Finally, in the abnormally structured anterior region (Lobules V–I) the IGL is missing and the PCs are ectopic. In addition, the IGL is missing and the PC are ectopically localised (abbreviations: EGL = External granular layer, PC = Purkinje cells, IGL = internal granular layer, Pf = primary fissure). Lobules are indicated by roman numerals I–X. Scale bar: E15,5: 100 µm; E18,5, P1: 200 µm; P7, P21: 0,5 mm.

**Figure 4:**
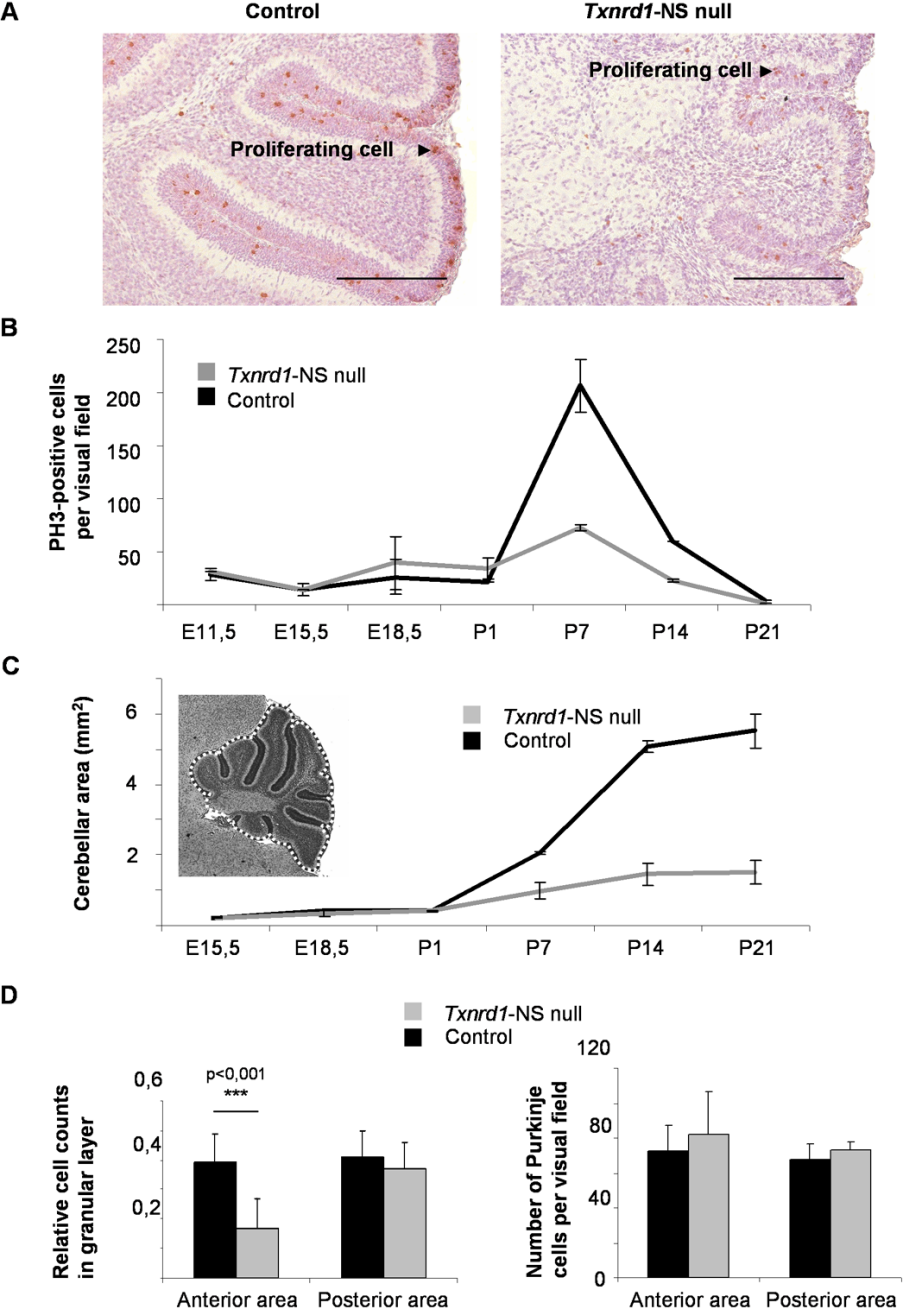
Reduced proliferation in *Txnrd1* null cerebellum. (A) To visualize mitotic cells, immunohistochemial staining on paraffin-embedded sagittal sections of P7 cerebelli were performed with an anti-phosphorylated histone 3 (PH3) antibody. Sections were counterstained with Mayer's haemalaun. Scale bar: 200 µm. (B) Illustrated is the number of PH3-positive cells per visual field from E11.5 until completion of postnatal cerebellar development at P21. (C) *Txnrd1*-NS null mice show a reduced cerebellar size from P1 onwards. Dashed line indicates the measured area. (D) Adult null mice show a strongly reduced relative cell density of the granular layer in the anterior cerebellum and only a slight reduction in the posterior part. The number of Purkinje cells remained unaffected.

While most brain regions, including the olfactory bulb, subventricular zone and the cerebral cortex, did not reveal any histological abnormalities in *Txnrd1*-NS null mice, the cerebellar cortex displayed striking morphological alterations. In contrast, no morphological abnormalities could be detected in *Txnrd2*-NS null mice, indicating that Txnrd1, rather than Txnrd2, plays a vital role for brain development. Further histological analyses were performed in *Txnrd1*-NS null mice to define the developmental abnormalities in the cerebellum. For a thorough spatiotemporal analysis of the cerebellar phenotype, serial sagittal sections of brains from control and *Txnrd1*-NS null mice at different embryonic stages (E11.5, E15.5 and E18.5) and various postnatal stages were prepared (Fig. 3). Cerebellar dimensions were determined morphometrically (Fig. 4). At developmental stages E11.5 (data not shown) and E15.5, no differences between knockout and control animals were evident. Overall cerebellar size and external granular layer (EGL) thickness were indistinguishable among mice of the different genotypes. From as early as E18.5 until the end of cerebellar development at postnatal stage P21, cerebellar morphology of the mutants substantially differed from that of control animals, particularly in its size, cerebellar cortical folding and lamination. Cerebellar size differences became evident from P7 onwards (Fig. 3 and Fig. 4 C). At weaning, the area of the knockout cerebellum was only one fourth of that of the controls. Massive proliferation within the EGL, producing granule neurons, is mainly responsible for the postnatal expansion of the cerebellum [21]. In normal development, it comprises 7–10 cell layers at P7, decreasing thereafter, and eventually disappears by P21 [22]. However, from P1, the EGL in the anterior cerebellum of the mutants was thinner than in control animals (Fig. 3), resulting in strongly diminished expansion of the tissue in this area.

In order to delineate the mechanism, underlying cerebellar hypoplasia in *Txnrd1*-NS null mice, proliferation of granule cell precursors was quantified by immunostaining for mitotic cells using the mitosis marker phosphorylated histone H3 (PH3) [23] (Fig. 4 A and B). In control mice, EGL proliferation was highest at postnatal day 7, whereas in *Txnrd1*-NS null mice the number of proliferating cells was approximately three times lower at P7, and around two times lower at P14 compared to control mice. The strongly impaired proliferation eventually resulted in a strongly reduced cell density of the granular layer particularly in the anterior part of the knockout cerebellum (Fig. 4 D). This effect was limited to granular cells, as the number of Purkinje Cells was unaffected (Fig. 4 D). To examine whether increased programmed cell death may contribute to the dramatically reduced size of the cerebellum during development, sagittal sections of P7 cerebella from *Txnrd1*-NS null mice and controls were stained for apoptotic cells [24]. However, no difference in the ratio of TUNEL-positive cells was apparent between control and mutant mice, indicating that increased cell death does not substantially contribute to impaired cerebellar size (Fig. S2).

Owing to the massive proliferation of granule cell precursor cells, the cerebellar cortex expands, finally developing into a folded structure with 10 main vermal lobules (designated I to X). Cerebellar development is completed by P21. In *Txnrd1*-deficient brain, the absence of major proliferation in the anterior region of the cerebellum leaves this folding unnecessary, and thus only lobules VII to X are formed (Fig. 3).

**Figure 5:**
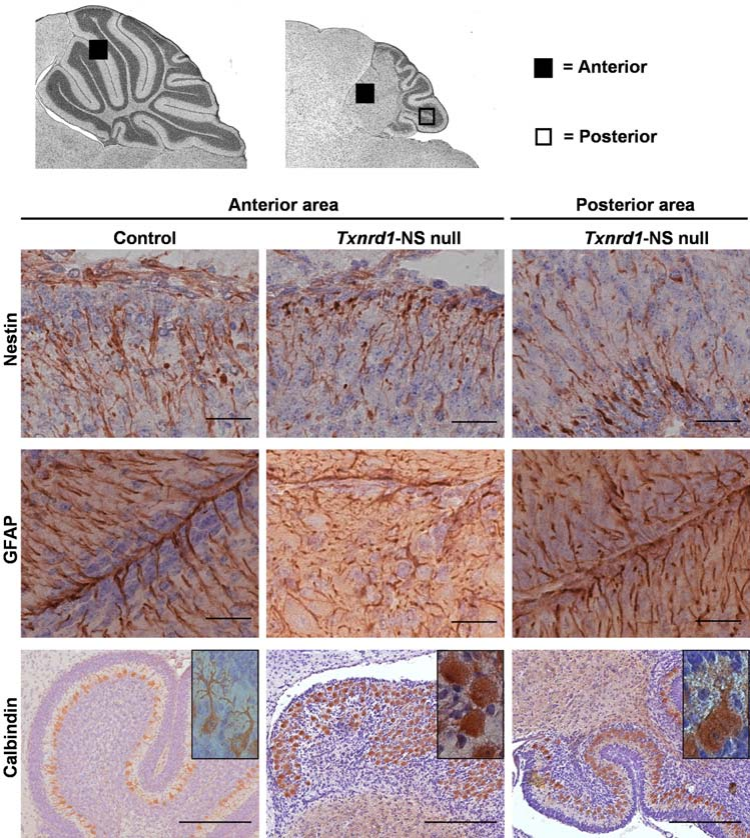
Glial disorientation, lobular fusion and ectopic localisation with reduced arborisation of Purkinje cells in anterior cerebellum of *Txnrd1*-NS null mice. Immunohistochemical staining of paraffin-embedded sagittal cerebellar slides using anti-Nestin (P1), anti-GFAP, and anti-Calbindin (both P14) counterstained with Mayer's haemalaun. The anterior and posterior cerebellar areas, represented in the high power micrographs shown in the lower part of the figure, are indicated by a filled box or an open box, respectively. Bergman Glia, shown as anti-Nestin or anti-GFAP immunoreactive cells, is essential for neuronal migration during pre- and postnatal cerebellar development. Clew-like alignment and shortening of Bergman glia is found in the anterior cerebellum of the mutant in 1 day old mutants (upper centre) or 14 day old mutants (middle centre). Anti-GFAP immunoreactive Bergmann glial fibers are shorter, disoriented, and reduced in density in the affected anterior *Txnrd1*-NS null lobules compared to control animals which show a radial alignment of these cells towards the pial surface (middle left). Glial cells in the posterior area of the *Txnrd1*-NS null cerebellum appear normal (upper and middle right). Purkinje cells, which are immunoreactive with anti-Calbindin, are located in a monolayer and project their dendrites towards the cells of the molecular layer in control mice (lower left). In the dysmorphic anterior *Txnrd1*-NS null cerebellum, ectopic Purkinje cell bodies in numerous layers fill the merged lobules and show impaired dendritic arborisation (lower centre). Purkinje cells of the mutant posterior cerebellar area show the same features, although less pronounced (lower right). Scale bar: 25 µm, anti-Calbindin staining: 200 µm.

Besides the differences in size and foliation, there was a striking lamination defect in the anterior part of the *Txnrd1*-deficient cerebellum. The definitive cerebellar cortex consists of a molecular layer, a Purkinje cell (PC) layer and an internal granular layer. PCs, originating from the ventricular zone, migrate along the Bergmann glial fibers to settle in a dense zone beneath the forming EGL. Terminal differentiation of PCs occurs postnatally when they become arranged in a monolayer and make contact with the parallel fibers of the granule cells [25]. The granular neurons forming the internal granular layer descend from the EGL after exiting the cell cycle and move radially from the outer cerebellar surface beneath the PC layer along the guiding Bergmann glia, thereby making their connections with the PC dendrites. These developmental events were severely disturbed in the cerebellum of the mutants and the anterior part became progressively more distorted throughout postnatal development (Fig. 3). The anterior cerebellum showed an ectopic PC localisation (Fig. 3, P7), no formation of distinctive molecular-, PC- and granular layer and fusion of the lobules. In the posterior part, however, lamination did not grossly deviate from the normal cerebellar development. To determine the roles of different cell types in the lamination phenotype, we performed immunohistochemical stainings (Fig. 5) [26]–[28]. By P1, staining for Nestin (Fig. 5) and GFAP (data not shown) revealed a clew-like alignment and shortening of Bergman glia in the anterior cerebellum of the mutant. By P14, a significant change in the distribution the GFAP-protein in the anterior mutant cerebellum, loss of radial alignment and reduction of thickness were detectable (Fig. 5). This was not observed in the posterior area of *Txnrd1*-NS null mice.

Immunostaining with the PC marker Calbindin (Fig. 5) clearly supports the ectopic localisation of PCs in the fused anterior cerebellar area, confirming the data already obtained by HE staining (Fig. 3, P7). In addition, architecture of the dendritic trees in the mutants was less elaborated (Fig. 5, inlays). PC dendritic trees along with the parallel fibers of granular neurons as well as basket- and stellate cells predominantly form the molecular layer, which does not separate in the anterior part of the mutants (Fig. 3 and Fig. 5). Immunostaining for parvalbumin, another marker for Purkinje cells, corroborated these findings (data not shown). Postmitotic neurons of the granular layer were detected by NeuN expression (data not shown) [29]. This staining supported the disrupted formation of the granular layer in the mature anterior cerebellum of *Txnrd1*-NS null mice as also revealed by the H&E staining (Fig. 3). In summary, the development of cerebellar lobules was defective in *Txnrd1*-NS null mice, showing fusion of lobule I–VI. In this anterior part, the principal laminae, molecular layer, PC layer, and granule layer, did not form. The lobules in the posterior cerebellar area (lobules VI–X) of *Txnrd1*-NS null mice were smaller but the laminar organisation was normal.

**Figure 6:**
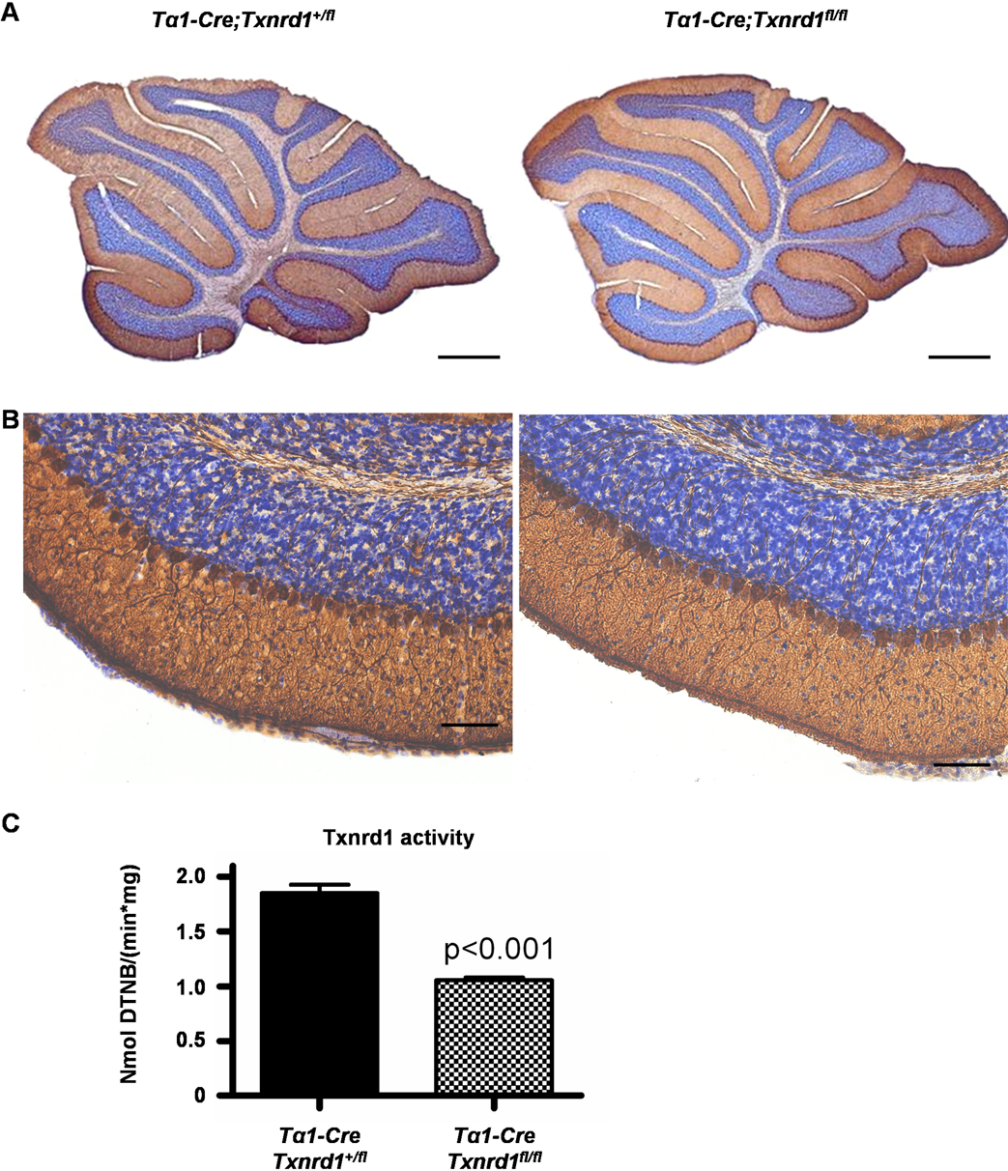
Mice with neuron-specific disruption of *Txnrd1* are phenotypically indistinguishable from wild-type litter mates. (A) Histological analysis of cerebellum revealed normal size and foliation pattern of the cerebellum of *Tα1-Cre;Txnrd1* ^fl/fl^ mice (with neuron-specific disruption of Txnrd1, right panel) in comparison to control mice (left panel) at the age of 5 weeks. Purkinje cells are stained with an antibody against Calbindin. (B) Higher power magnifications from (A). Sections were counterstained with Nissl. (B) Reduced cytosolic thioredoxin reductase activity in neuron-specific *Txnrd1* knockout mice. Residual activity most likely resides in glial cells. Scale bars: 250 µm

In order to further dissect the roles of neurons and glial cells in Txnrd1-dependent developmental processes, we additionally generated neuron-specific *Txnrd1* knockout mice (*Tα1-Cre;Txnrd1^fl/fl^*) [30], [31]. Interestingly, cerebellar foliation was completely normal in these mice (Fig. 6 A, B). Accordingly, there were no behavioural abnormalities or growth defects in these mice (not shown). Cytosolic thioredoxin reductase activity measurements were performed to demonstrate targeted inactivation of Txnrd1 in neuronal cells (Fig. 6 C). Thioredoxin reductase activity was reduced by half, consistent with remaining Txnrd1 expression in glial cells. Thus, since Nestin-Cre causes excision of loxP flanked genes in neuronal and glial precursor cells, but *Tα1-Cre* mice are specific for postnatal neurons, it is tempting to assume that the cerebellar phenotype in *Txnrd1*-NS null mice is caused by disruption of Bergmann glia function and/or diminished proliferative capacity of granule cell precursors.

## Discussion

The thioredoxin (Txn) dependent system, comprising of cytosolic (Txn1) and mitochondrial (Txn2) thioredoxins, various Txn dependent peroxidases (peroxiredoxins) and the selenoenzymes thioredoxin reductases 1–3, is one of the major redox systems in cells involved in the control of cellular redox balance [2], [8]. While the Txn dependent system has been suspected to play a significant role in brain tissue, only very little information on its physiological role in brain development and function has been available to date.

To address the individual roles of members of the thioredoxin reductase family in brain development and function, we conditionally disrupted *Txnrd1* and *Txnrd2* expression in brain using neural-specific and neuron-specific Cre transgenic mice. Thus, expression of *Txnrd1* and *Txnrd2* is abrogated as early as in neuronal progenitor cells [18] or early postnatal neurons [30]. Cre-mediated inactivation was highly efficient in *Txnrd1*- and *Txnrd2*-NS null mice. While expression levels of *Txnrd1* were very low in the brain of *Txnrd1*-NS null mice, residual cytosolic thioredoxin reductase activity could be detected in these mice, which may be attributed to cytosolic Txnrd2. Several recent reports indicated that *Txnrd2* is also expressed in the cytosolic compartment [11], [32], though its subcellular localisation in tissues and organs remains to be elucidated. Surprisingly, mice specifically lacking Txnrd2 in the NS develop normally, do not show any histopathological deterioration and have a similar life span as control littermates (data not shown). By contrast, NS-specific *Txnrd1* knockout mice exhibit reduced weight gain from P7 onwards and developed severe ataxia, suggestive of major cerebellar defects. Nonetheless, NS-specific *Txnrd1* knockout mice survived and could even generate and foster viable offspring. Our data imply that it is Txnrd1 and not Txnrd2 that plays a critical role in brain development, however this does not rule out any contribution of Txnrd2, for instance under pathological conditions such as stroke and trauma. Interestingly, in our two previous studies we could clearly assign a crucial role for Txnrd2 in heart development and function [16], while Txnrd1 function turned out to be negligible for cardiac development and function [17]. Thus, both enzymes do not emerge as mere housekeeping genes, but fulfil tissue and organ-specific functions.

We observed a clear compartment boundary between the widely normally developed posterior cerebellum (lobules VII–X) and the strongly disorganized anterior part (lobules I–VI) in *Txnrd1*-NS null mice. This phenomenon has been previously found in a number of other cerebellar aberrations [33]–[35], and has been interpreted as a general developmental principle in cerebellar development [34]. Most interestingly, loss-of genetic loci causes a phenotype either in the anterior or the posterior part of the cerebellum. Meander tail (Mea) for example has an appearance that very closely resembles the *Txnrd1*-NS null phenotype, whereas *BETA2/NeuroD1*-knockout has a posterior defect [36]. In contrast to our findings, the cell types primarily affected by the Mea mutation are the granular neurons. The molecular and cellular mechanisms leading to the Mea phenotype, however, have not been described yet.

Histopathological analysis of *Txnrd1* null brain revealed striking cerebellar hypoplasia, in particular of lobules I–VI. Proper foliation requires a complex, well-coordinated series of developmental steps, including proliferation or death of granule cells [37], migration along Bergmann glial fibers [38] and outgrowth of PC dendrites [22]. All these processes are intricately connected [39], e.g. granule cell precursor proliferation depends on sonic hedgehog released by PC [40], and induction of a radial glial phenotype of Bergmann glia requires the release of Neuregulin by granule cells. Since increased cell death is not observed, but reduced numbers of mitotic granule cell precursors, the major defect underlying cerebellar hypoplasia might result from diminished proliferation of granule cells, the driving force of cerebellar growth and foliation. In fact, it has been demonstrated recently that increasing sonic hedgehog signalling in the mouse cerebellum (by modulating patched expression) can induce the formation of an additional lobe at the same position where it exists in rats [40]. A major role for Txnrd1 in postnatal proliferation of granule cells is also suggested by the normal embryonic development of the cerebellum. The use of two different Cre-transgenic mice to disrupt *Txnrd1* allowed us to rule out a major role of neuronal Txnrd1 for cerebellar morphogenesis. Thus, either radial glial (Bergmann) cells and/or granule cell precursors are the cell type likely affected by Txnrd1-deficiency.

Interestingly, neither the cerebellar anlage nor any other part of the brain seems to be affected by loss-of *Txnrd1* using NesCre. This is a remarkable finding, since virtually all neurons show Nestin expression during their development. This finding is underscored by the complete absence of Txnrd1 protein observed in brain homogenates from *Txnrd1-*NS null mice. So why is the cerebellum apparently more affected than other brain regions that also descend from proliferation of neuroepithelial cells? If we assume the granule cell precursors as the primarily affected cell type, they are distinguished from most other proliferative epithelia in being a secondary neuroepithelium. There is, however, no indication why a secondary neuroepithelium should specifically rely on Txnrd1. Another difference is the massive proliferation, occurring during postnatal cerebellar expansion. In fact, granule cells are the single most abundant cell type in the brain. Thus, if Txnrd1 is absent, insufficient provision of electrons for ribonucleotide reductase [41], [42] (the commited step in DNA synthesis) during massive expansion might become the limiting step in tissue development.

Of interest, neuronal progenitor cell-specific ablation of *N-myc* using the *Nestin-Cre*, resulted in a similar cerebellar phenotype as in the like NS-specific *Txnrd1* knockout mice [43]. NS-specific *N-myc* null mice show ataxia and behavioural abnormalities, associated with a strong reduction in cerebellar size. Drops in mitotic cells and cells in S-phase along with reduced expression of the N-myc target gene *cyclin D2* were considered being responsible for cerebellar hypoplasia in *N-myc* null brain. *Txnrd1* has been identified as direct c-Myc target gene in a human B cell line [44]. Since members of the Myc transcription factor family are largely interchangeable [45], and n-Myc constitutes the major isoform of the Myc family in brain, one may propose that *Txnrd1* is one of the most significant target genes of n-Myc in brain. Also, the Txn1/Txnrd1 system has been frequently linked with proliferation of many tumors and tumor cell lines [46]–[48]. Thus, our present finding along with our previous studies, in which we observed a pronounced overall proliferation defect in *Txnrd1* null embryos except in the heart [17], corroborate the importance of Txnrd1 for cell proliferation and tissue development. It is, however, somewhat surprising that other regions of the brain apparently do not require functional Txnrd1 or Txnrd2. It thus remains open, whether thioredoxin reductase 3 (also called thioredoxin-glutaredoxin reductase) may replace Txnrd1 or Txnrd2 [11], or alternatively that Txnrd2 may functionally compensate for dysfunctional Txnrd1 at least for normal proliferation. This possibility might be answered by generating compound mutant mice in which both isoforms are simultaneously inactivated in the brain. Of note, knockout mouse models for enzymes of the cell cycle progression machinery, including *cyclin D2* [49] or compound mutant mice lacking cyclins D1 and D2 [50] also develop similar phenotypes in the cerebellum, albeit to a different degree. Hence, Txnrd1 might be viewed as an additional major player of the cell proliferation machinery.

Recent data implied that truncated forms of Txnrd1, lacking the C-terminally located catalytically important selenocysteine might exert pro-apoptotic functions [51], [52]. Since our targeting strategies aimed at inactivating the C-terminal catalytic centers of Txnrd1 and Txnrd2, we cannot rule out that minute amounts of Txnrd1 or Txnrd2 are still made in knockout brains, possibly causing unwanted side effects. But in our NS-specific knockout models, we could never detect any changes in the number of apoptotic cells, even in aged brain (data not shown).

Selenium depletion experiments in rodents demonstrated that selenium is strongly retained in brain, suggesting vital functions for one or several selenium dependent enzymes for neuronal and non-neuronal cells. Accordingly, mice with targeted disruption of selenoprotein P, a selenoprotein of the selenium delivery pathway to organs, show seizures and ataxic gait [13]. Neuron-specific ablation of selenoprotein expression, achieved by neuron-specific removal of the tRNA for selenocysteine, leads, among other phenotypes, to cerebellar hypoplasia. However, the defect is histologically distinct from that observed in NS-specific *Txnrd1* knockout mice (E. Wirth U.S., unpublished).

The Txn1-Txnrd1 system has been implicated in Alzheimer's disease. Altered expression levels have been observed in patients suffering from Alzheimer's disease, and pre-treatment of primary hippocampal cultures with either Txn or Txnrd appear to ameliorate the effects of amyloid *β* [53]. Likewise, oxidation of glutaredoxin 1 and thioredoxin 1 in response to amyloid *β* treatment has been shown to activate apoptosis signal-regulating kinase (ASK1), suggesting that a deregulated antioxidant system including thioredoxin 1 might be critical in the sequelae leading to Alzheimer [54]. In future experiments, our models might prove most suitable to investigate the role of the Txn1/Txnrd1 system in animal models of Alzheimer's Diseases or other neurodegenerative diseases.

## Materials and Methods

### Generation and maintenance of tissue-specific knockout mice

To generate neuronal progenitor cell-specific or neuron specific thioredoxin reductase-deficient mice, mice with conditional alleles for either *Txnrd1* (*Txnrd1* ^tm1Marc^) [17] or *Txnrd2* (*Txnrd2* ^tm1Marc^)[16] were crossed with Nestin-Cre transgenic mice [18] or *Tα1-Cre* mice, respectively [30]. Two consecutive breeding steps were required. First, loxP-flanked (floxed; fl) *Txnrd1* ^tm1Marc^ (designated as *Txnrd1* ^+/fl^) and Txnrd2^tm1Marc^ (designated as *Txnrd2* ^+/fl^) knockout mice were bred with *Nestin-Cre* or *Tα1-Cre* transgenic mice to obtain *NesCre;Txnrd1* ^+/fl^, *Tα1-Cre;Txnrd1* ^+/fl^, and *NesCre;Txnrd2* ^+/fl^ mice, respectively. Subsequently, these mice were mated with *Txnrd1* ^fl/fl^ or *Txnrd2* ^fl/fl^ mice to obtain *NesCre;Txnrd1* ^fl/fl^ (designated as *Txnrd1*-NS null mice in the text), *Tα1-Cre;Txnrd1* ^fl/fl^ (designated as neuron-specific *Txnrd1* knockout mice), and *NesCre;Txnrd2* ^fl/fl^ mice (designated as *Txnrd2*-NS null mice in the text), respectively. Heterozygous knockout mice behaved like wild-type in all assays, and thus *NesCre;Txnrd1* ^+/fl^, *Txnrd1* ^fl/fl^, *Txnrd1* ^+/fl^ and *NesCre;Txnrd2* ^+/fl^, *Txnrd2* ^+/fl^, *Txnrd2* ^+/fl^ littermates served as controls. Mice were kept under standard housing conditions with food (type 1314, Altromin GmbH, Lage, Germany) and water ad libitum. All animal experiments were performed in compliance with the German animal welfare law and have been approved by the institutional committee on animal experimentation and the district governments of Upper Bavaria and Berlin. Genotyping of mice was performed by PCR of tail DNA as described previously [16], [17].

### Immunoblotting

Preparation of total and cytosolic protein fractions from tissue was carried out as described [17]. Protein concentrations were determined by the Bradford assay (Bio-Rad Labs GmbH, Munich, Germany). 20 µg of cytosolic proteins were separated on a 10% SDS-PAGE and blotted onto a nitrocellulose membrane (Hybond ECL, Amersham Biosciences, RPN 303D). For detection, a rabbit anti-Txnrd1 (1∶2000 dilution, Lab Frontier, LF-PA0023, Seoul, Korea), and a goat anti-rabbit horseradish peroxidase conjugated secondary antibody (1∶2000 dilution, Bio-Rad Labs GmbH, Munich, Germany) were used. Signals were visualized by an enhanced chemoluminescence kit (Amersham Biosciences, RPN 2106).

### RNA isolation, cDNA synthesis and quantitative RT-PCR (qRT-PCR)

Isolation of total RNA, DNAse treatment of RNA and reverse transcription was carried out as described [16]. qRT-PCR was carried out using a Roche LightCycler ® 2.0 Real-Time PCR System according to the manufacturer's recommendations (Roche Diagnostics, Mannheim, Germany). *Txnrd2* levels were normalized against 18S ribosomal RNA. Primers used for *Txnrd2* quantification were: E6/E10forw.: 5′-CAGCTTTGTGGATGAGCACACAGTTCG-3′, E6/E10rev.:5′-GATCCTCCCAAGTGACCTGCAGCTGG-3′, E15/E18forw.:5′-TTCACGGTGGCGGATAGGGATGCTC-3′, E15/E18rev 5′-TGCCCAGGCCATCATCATCTGACG-3′, S18forw.:5′-GGA CAG GAT TGA CAG ATT GAT AG-3′, S18rev.: 5′-CTC GTT CGT TAT CGG AAT TAA C-3′.

### Determination of thioredoxin reductase activity

Thioredoxin reductase activity was determined by the NADPH-dependent DTNB reduction assay (Holmgren and Bjornstedt, 1995). 100 µg of the cytosolic fraction was added to the reaction mixture, containing 0.1 M potassium phosphate, pH 7.0, 1 mM EDTA, 2 mg/ml DTNB (Sigma-Aldrich GmbH, Seelze, Germany), 0.2 mg/ml NADPH (10107824001, Roche Diagnostics, Mannheim, Germany), and 0.2 mg/ml bovine serum albumin. Absorption at 412 nm was measured in a spectrophotometer (SmartSpec ™ Plus, BioRad GmbH, Munich, Germany). Txnrd1 activity is expressed as nmol oxidized NADPH/(min * mg of protein).

### Pole test

The pole test was performed according to Fernagut et al. [20], with minor modifications. 14 *Txnrd1*-NS null and 14 control littermates were placed head downwards on top of a vertical rough-surfaced pole (diameter 1 cm, 50 cm height). Nesting material was placed at the bottom of the pole to avoid injury. Each mouse was habituated to the pole on the day prior to testing (five trials). The total time until the mouse reached the floor with its four paws was recorded (T-total). Animals received five trials with an inter-trial interval of 30 s. If the mouse was unable to climb down, fell or slipped, a default value of 120 s was taken into account.

### Histological analysis

Embryos dissected at various developmental stages (E11.5, E15.5, E18.5) as well as brains from animals at postnatal stages P1, P7, P14, P21 and two years old were analyzed. Freshly dissected embryos and tissue samples were fixed overnight in 4% (w/v) paraformaldehyde (4% PFA) in phosphate buffered saline (PBS) or in Bouin's fixative, stored in 70% ethanol, embedded in paraffin, and cut in sections of 7 µm (embryos) and 8 µm (brains). For hematoxylin-eosin (HE) staining, sections were deparaffinized in xylol, rehydrated in graded ethanol series, stained with Mayer's haemalaun (Carl Roth, Karlsruhe, Germany) for 5 min, washed again, and stained with 1% eosin (Carl Roth, Karlsruhe, Germany for 2 minutes). The sections were washed in water, dehydrated in graded ethanol series, treated with xylol, and mounted. Nissl staining was carried out as described [55].

### Immunohistochemical stainings

Immunohistochemistry on paraffin-embedded sections was carried out as described previously [16], [17]. Following primary antibodies were used: anti-phosphorylated histone H3 (PH3) (9701, Cell Signaling NEB GmbH, Frankfurt, Germany), anti-GFAP (MAB360), anti-calbindin (AB1778), anti-Nestin (MAB353), anti-Parvalbumin (MAB 1572) and anti-NeuN (MAB377, all Chemicon GmbH, Schwalbach, Germany). Biotinylated secondary antibodies were purchased from Vector labs, Ohio, USA. To visualize bound secondary antibodies, staining was performed according to the manufacturer's protocol (Vectastatin Elite ABC kit PK-6100 and DAB kit SK-4100, Vector Labs. Ohio, USA). Sections were counterstained with Mayer's haemalaun (Carl Roth, Karlsruhe, Germany). To avoid non-specific binding 5% BSA/PBS-solution (MP Biomedicals) was used for blocking.

### In situ end labelling

For monitoring apoptotic cells in *Txnrd1*-NS null and control animals, PFA-fixed (4% (w/v) paraformaldehyde in PBS) and paraffin-embedded sections were stained with the ApopTag kit (S7100, Serologicals Corporation, Norcross, GA, USA) according to the manufacturer's instructions. Digoxigenin-deoxynucleoside triphosphate-labelled cells were incubated with an anti-digoxigenin peroxidase conjugate and visualized with 3,3′-diaminobenzidine (DAB, Vector Labs, Ohio, USA). Sections of testis from *C57Bl/6* animals served as positive control.

### Morphometrical and quantitative analyses

Documentation of the sections was performed using an Axioplan 2 microscope and Axiovision 3.1 software (Zeiss, Oberkochen, Germany). Mitotic cells (PH3-positive cells): The number of PH3-positive cells in the cerebellum per section were counted on sections from two *Txnrd1*-NS null and two control animals at developmental and neonatal stages E11.5, E15.5, E18.5, P1, P7, P14, and P21. Cerebellar area: Measurement of the cerebellar area in midsaggital sections of two *Txnrd1*-NS null and control animals was performed by using the Axiovision LE 4.1 software (Zeiss, Oberkochen, Germany) at stages E15.5, E18.5, P1, P7, P14, and P21. Relative cell counts in the cerebellar granular layer and number of Purkinje cells1: Quantification of cells in the anterior and posterior part of the cerebellum (three knockouts and three controls, adult animals) was calculated as described previously (Leung et al., 2004), using the ImageJ program (http://www.uhnresearch.ca/facilities/wcif/imagej/) for granular cells. Purkinje cells were counted directly on the microscope.

## Supporting Information

Figure S1 *Txnrd2* is dispensable for brain development. (A) Quantitative RT-PCR analysis of total brain RNA revealed that *Txnrd2* transcripts were strongly reduced in *Txnrd2*-NS null mice (empty bars) compared to control mice (filled bars). Two primer pairs, one specifically detecting the core region of *Txnrd2* (E6/E10) and one specific for the deleted region (E15/E18) were used. *Txnrd2* expression was normalized to 18S rRNA expression levels (shown is the mean +/− SD) (B) H&E staining of mid-sagittal cerebellar sections from adult control (left column) and *Txnrd2*-NS null mice (right column). The cerebellum, like other brain regions (data not shown), did not display any defects in lamination and foliation. Scale bars: 250 μm. (C) TUNEL staining of brain sections did not show any increases in the number of apoptotic cells in the cerebellum of *Txnrd2*-NS null brain. Germinal epithelium of testicular tissue served as a positive control (inlay). Scale bars: 50 μm. Cerebellar lobules are indicated by roman numerals I–X.
(2.53 MB TIF)

Figure S2 Apoptotic cell numbers are unaltered in the *Txnrd1*-NS null cerebellum. (A) Apoptotic cells (arrowheads) are commonly found in the germinal epithelium in testis, which served as a positive control. (B) Negative control without primary antibody: no imunoreactivity with the secondary antibody. Illustrated are the anterior cerebellar regions from a control mouse (C) and a *Txnrd1*-NS null mouse (D). Arrowheads show apoptotic cells in the EGL. There is no increased level of apoptosis in the cerebellum of the mutants. Abbreviations are as follows; SC: Sertoli cell; SpG: Spermatogonium; PrS: Primary spermatocytes; SpT: Spermatids. EGL: External granular layer; IGL: Internal granular layer; PC: Purkinje cell layer. Arrowheads indicate apoptotic cells. Scale bars: 50 μm.
(2.00 MB TIF)
