# Supplementary figures and images for: The Role of Thioredoxin Reductases in Brain Development
Source: PLoS One. 2008 Mar 19;3(3):e1813. doi: 10.1371/journal.pone.0001813 (PMC2263137; doi:10.1371/journal.pone.0001813)

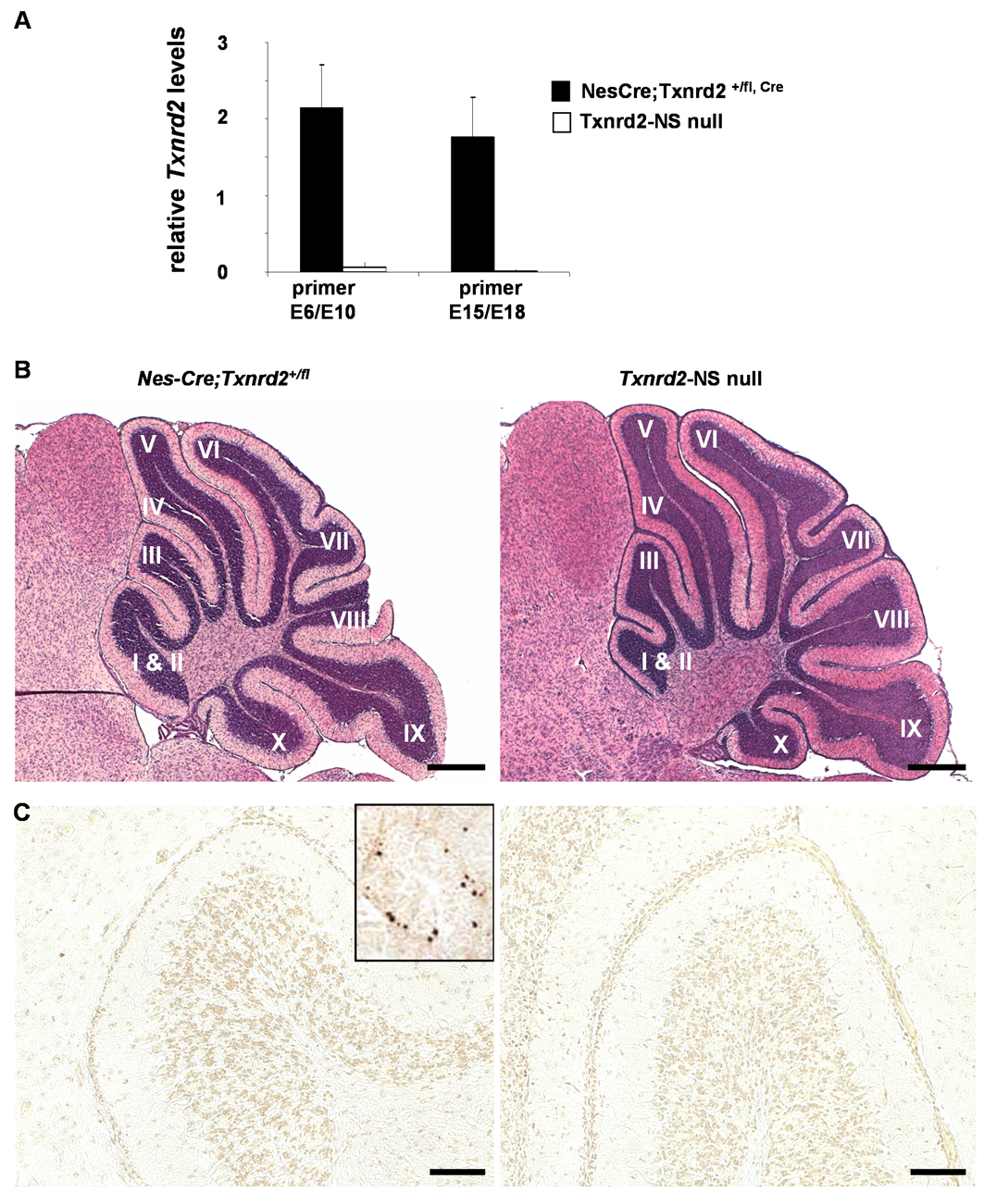

Supplement: Figure S1 — Txnrd2 is dispensable for brain development. (A) Quantitative RT-PCR analysis of total brain RNA revealed that Txnrd2 transcripts were strongly reduced in Txnrd2-NS null mice (empty bars) compared to control mice (filled bars). Two primer pairs, one specifically detecting the core region of Txnrd2 (E6/E10) and one specific for the deleted region (E15/E18) were used. Txnrd2 expression was normalized to 18S rRNA expression levels (shown is the mean +/− SD) (B) H&E staining of mid-sagittal cerebellar sections from adult control (left column) and Txnrd2-NS null mice (right column). The cerebellum, like other brain regions (data not shown), did not display any defects in lamination and foliation. Scale bars: 250 μm. (C) TUNEL staining of brain sections did not show any increases in the number of apoptotic cells in the cerebellum of Txnrd2-NS null brain. Germinal epithelium of testicular tissue served as a positive control (inlay). Scale bars: 50 μm. Cerebellar lobules are indicated by roman numerals I–X. (2.53 MB TIF) [file pone.0001813.s001.tif]

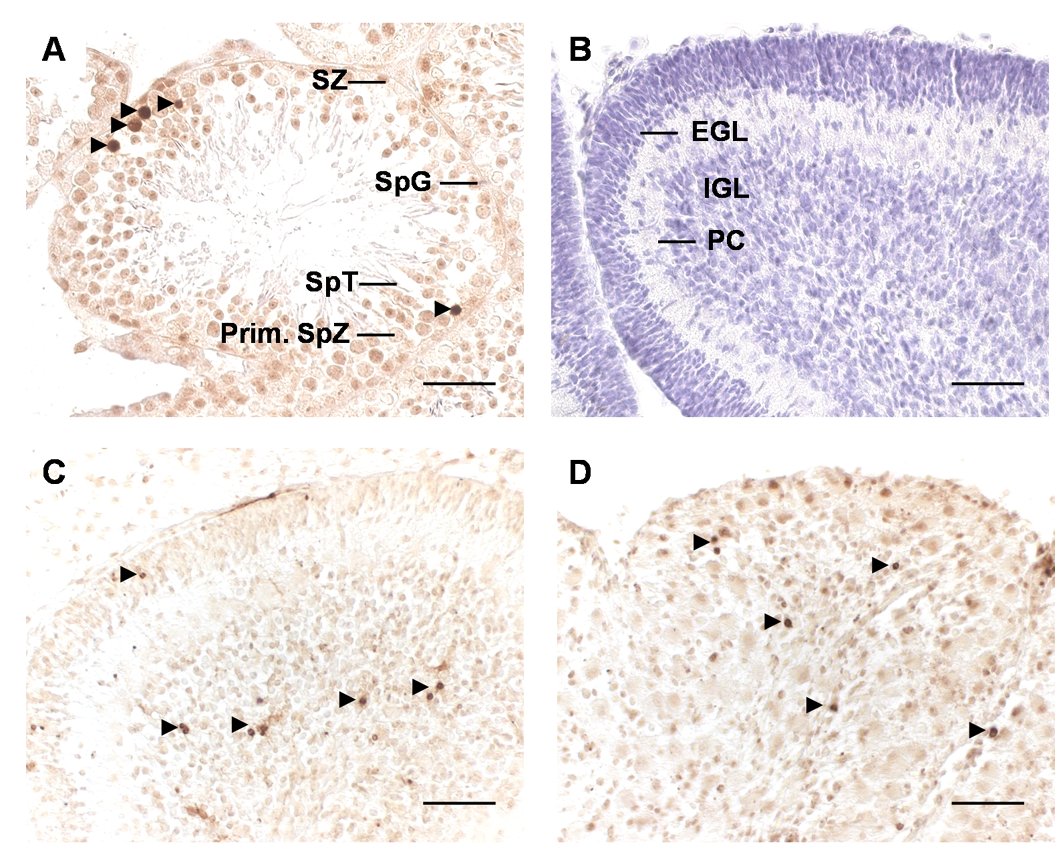

Supplement: Figure S2 — Apoptotic cell numbers are unaltered in the Txnrd1-NS null cerebellum. (A) Apoptotic cells (arrowheads) are commonly found in the germinal epithelium in testis, which served as a positive control. (B) Negative control without primary antibody: no imunoreactivity with the secondary antibody. Illustrated are the anterior cerebellar regions from a control mouse (C) and a Txnrd1-NS null mouse (D). Arrowheads show apoptotic cells in the EGL. There is no increased level of apoptosis in the cerebellum of the mutants. Abbreviations are as follows; SC: Sertoli cell; SpG: Spermatogonium; PrS: Primary spermatocytes; SpT: Spermatids. EGL: External granular layer; IGL: Internal granular layer; PC: Purkinje cell layer. Arrowheads indicate apoptotic cells. Scale bars: 50 μm. (2.00 MB TIF) [file pone.0001813.s002.tif]
